# Prognostic Roles of ceRNA Network-Based Signatures in Gastrointestinal Cancers

**DOI:** 10.3389/fonc.2022.921194

**Published:** 2022-07-18

**Authors:** Xin Qi, Xingqi Chen, Yuanchun Zhao, Jiajia Chen, Beifang Niu, Bairong Shen

**Affiliations:** ^1^ School of Chemistry and Life Sciences, Suzhou University of Science and Technology, Suzhou, China; ^2^ Computer Network Information Center, Chinese Academy of Sciences, Beijing, China; ^3^ University of Chinese Academy of Sciences, Beijing, China; ^4^ Institutes for Systems Genetics, Frontiers Science Center for Disease-related Molecular Network, West China Hospital, Sichuan University, Chengdu, China

**Keywords:** ceRNA network, prognostic signature, gastrointestinal cancer, translational implication, lncRNA, circRNA

## Abstract

Gastrointestinal cancers (GICs) are high-incidence malignant tumors that seriously threaten human health around the world. Their complexity and heterogeneity make the classic staging system insufficient to guide patient management. Recently, competing endogenous RNA (ceRNA) interactions that closely link the function of protein-coding RNAs with that of non-coding RNAs, such as long non-coding RNA (lncRNA) and circular RNA (circRNA), has emerged as a novel molecular mechanism influencing miRNA-mediated gene regulation. Especially, ceRNA networks have proven to be powerful tools for deciphering cancer mechanisms and predicting therapeutic responses at the system level. Moreover, abnormal gene expression is one of the critical breaking events that disturb the stability of ceRNA network, highlighting the role of molecular biomarkers in optimizing cancer management and treatment. Therefore, developing prognostic signatures based on cancer-specific ceRNA network is of great significance for predicting clinical outcome or chemotherapy benefits of GIC patients. We herein introduce the current frontiers of ceRNA crosstalk in relation to their pathological implications and translational potentials in GICs, review the current researches on the prognostic signatures based on lncRNA or circRNA-mediated ceRNA networks in GICs, and highlight the translational implications of ceRNA signatures for GICs management. Furthermore, we summarize the computational approaches for establishing ceRNA network-based prognostic signatures, providing important clues for deciphering GIC biomarkers.

## Introduction

Gastrointestinal cancer (GIC), mainly including gastric cancer (GC), colorectal cancer (CRC) and esophagus cancer (EC), represents a common threat to public health, with morbidity and mortality accounting for more than 15% of all cancers (1). Although significant progress in treatment strategies, e.g. surgery, chemotherapy, targeted therapy and radiotherapy, has been achieved over the past years, the outcomes of GICs are still disappointing since they mostly develop with no obvious symptoms and are frequently diagnosed at advanced stages (2). Moreover, due to complexity and heterogeneity, GIC patients with identical pathologic conditions often exhibit huge variation in treatment response and prognosis, limiting the application of traditional approaches (e.g. tumor-node-metastasis (TNM) pathological staging) to distinguish patients at high risk of metastasis or death. Therefore, it is critical to develop novel and powerful prognostic models that can provide reliable information for patient risk stratification and treatment choice.

Early researches on the molecular mechanisms of tumorigenesis were mainly focused on the function of protein-coding genes, as proteins were traditionally considered as the central function executor. In the past two decades, the technological advances in next-generation sequencing approaches have enabled the system-level understanding of biological processes, which revealed that the presence of numerous non-coding RNAs (ncRNAs) contributes to the diversity and complexity of human transcriptome (3). Importantly, due to their regulatory roles in cellular events necessary for growth and development, ncRNA abnormal expression is closely linked to cancer pathogenesis (4, 5). Therefore, the exploration of ncRNAs can provide critical clues for identifying novel diagnostic and/or therapeutic targets in multiple cancer types.

ncRNAs comprise a diverse variety of RNA species, e.g. microRNA (miRNA), long noncoding RNA (lncRNA), circular RNA (circRNA) and etc. (6). Among them, miRNAs perform post-transcriptional regulatory roles by binding to miRNA-response elements (MREs) of target mRNAs (7). Increasing studies have demonstrated that target genes carrying common MREs can compete to sponge the same miRNA. Accordingly, competing endogenous RNAs (ceRNAs) hypothesis was put forward by Salmena et al. in 2011 (8) and has received extensive attention since then. It postulates that coding and non-coding RNA molecules with common MREs can compete for miRNA binding at these sites, thus indirectly regulating the expression of each other by acting as miRNA sponge (9).

Currently, as new functional players in cancer biology, lncRNA and circRNA have emerged as the most important ceRNA types (10, 11). Especially, based on the pivotal roles of ceRNA crosstalk in modulating cancer hallmarks, systematic construction and analysis of lncRNA/circRNA-mediated ceRNA network has recently become a powerful tool for decoding the underlying molecular mechanism of cancers and identifying prognostic biomarkers in these diseases (12, 13). Besides, many pseudogenes can also crosstalk with protein-coding genes by acting as ceRNAs to sequester shared miRNAs. For example, RP11-3543B.1 has been identified as an oncogenic pseudogene that implicated in GC pathogenesis by regulating MAPK4 expression *via* a ceRNA mechanism (14). However, there is little evidence for pseudogene-related prognostic signatures in GICs. Therefore, we here introduce the functional roles of lncRNA/circRNA-mediated ceRNA crosstalks in the pathogenesis of GICs, present a review on the prognostic signatures constructed based on lncRNA/circRNA-mediated ceRNA network in GICs, and summarize the computational strategy for establishing prognostic signatures based on ceRNA network.

## lncRNA/circRNA-Mediated ceRNA Crosstalks in GICs: Functional Roles and Prognostic Implications

As two novel classes of ncRNA regulators, lncRNAs and circRNAs play critical roles in multiple steps of cancer initiation and progression. With the innovations in biotechnology and bioinformatics, they are increasingly identified and characterized in GICs through genomic and transcriptomic studies (15, 16). Notably, given the ability to interact with miRNAs, both lncRNA and circRNA have emerged as the most important ceRNA players with prognostic significance in GICs.

**Figure 1:**
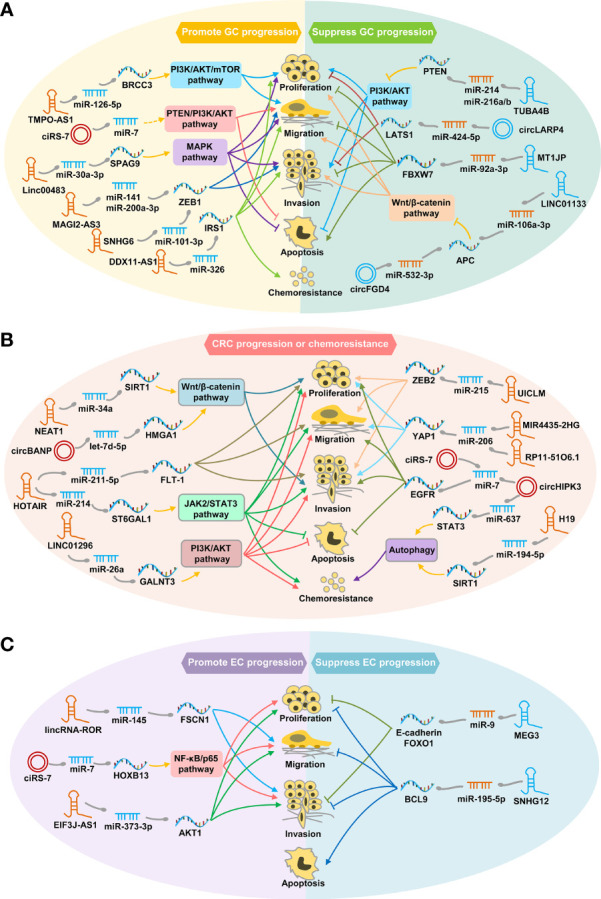
Schematic diagram of representative ceRNA crosstalks function in GC **(A)**, CRC **(B)** and EC **(C)**. **(A)** ceRNA interaction regulates tumor cell proliferation, migration, invasion, apoptosis, or chemoresistance through PI3K/ATK, MAPK or Wnt/β-catenin signaling pathways, thereby exerting carcinogenic or tumor suppressor effects in GC. **(B)** ceRNA interaction contributes to CRC progression or chemoresistance by regulating autophagy process or pivotal pathways, such as Wnt/β-catenin, PI3K/ATK and JAK2/STAT3 signaling pathway. **(C)** ceRNA interaction promotes or inhibits EC progression by modulating cancer cell proliferation, migration, invasion, or apoptosis.

Mounting evidence has demonstrated the profound impact of lncRNA/circRNA-mediated ceRNA interactions on multiple processes and events in the pathogenesis of GC, CRC and EC, such as cell proliferation, invasion, migration, apoptosis, or chemoresistance (**Figure 1**). For example, lncRNA MAGI2-AS3 can regulate the expression of epithelial-mesenchymal transition (EMT) transcription factor ZEB1 by sponging miR-141/200a to promote GC cell migration and invasion (17). By regulating the Wnt/β-catenin pathway, circFGD4 and LINC01133 serving as ceRNAs of APC, inhibit GC progression (18, 19), while circBANP and NEAT1-mediated ceRNA crosstalks contribute to CRC cell proliferation and invasion (20, 21).

Besides, lncRNA/circRNA-mediated ceRNA crosstalks are able to facilitate risk stratification and guide clinical decision-making for GIC patients (**Table 1**). For example, the small nucleolar RNA host gene (SNHG) family members (e.g. SNHG6, SNHG11 and SNHG12) are newly recognized important lncRNAs that promote tumor progression through the ceRNA mechanism (22–24). Increased SNHG6 expression was significantly correlated with poor prognosis of both GC and CRC patients (22, 25). In addition, it has been shown that ciRS-7 can act as an oncogene by inhibiting miR-7 activity *via* a ceRNA manner in GC, CRC and EC (**Figure 1**) (26–28), making it a promising prognostic biomarker and an attractive therapeutic target for GIC patients.

**Table 1:** Prognostic lncRNAs and circRNAs that function by a ceRNA mechanism in GICs.

ceRNA	Shared miRNA	Target mRNA	Clinical significance	Role in cancer	Cancer type	PMID
**lncRNA-mediated ceRNA crosstalk**						
XIST	miR-101	EZH2	Prognostic biomarker and therapeutic target	Promote	GC	27620004
LINC01939	miR-17-5p	EGR2	Prognostic biomarker and therapeutic target	Suppress	GC	30683847
LINC02163	miR-593-3p	FOXK1	Prognostic biomarker and therapeutic target	Promote	GC	29893595
CCDC144NL-AS1	miR-143-3p	MAP3K7	Therapeutic target	Promote	GC	32647147
TUBA4B	miR-214, miR-216a/b	PTEN	Prognostic biomarker and therapeutic target	Suppress	GC	31198405
TMPO-AS1	miR-126-5p	BRCC3	Therapeutic strategy	Promote	GC	33295056
ADPGK-AS1	miR-3196	KDM1B	Prognostic biomarker and therapeutic target	Promote	GC	30944080
FEZF1-AS1	miR-363-3p	HMGA2	Therapeutic target	Promote	GC	32638620
Lnc-ATB	miR-141-3p	TGFβ2	Prognostic predictor and therapeutic target	Promote	GC	28115163
MAGI2-AS3	miR-141/200a-3p	ZEB1	Biomarker and therapeutic target	Promote	GC	31837602
Linc00483	miR-30a-3p	SPAG9	Prognostic biomarker	Promote	GC	29761936
DLX6-AS1	miR-204-5p	OCT1	Prognostic biomarker and therapeutic target	Promote	GC	31463827
HOTAIR	miR-331-3p	HER2	Prognostic biomarker and therapeutic target	Promote	GC	24775712
BC032469	miR-1207-5p	hTERT	Prognostic biomarker	Promote	GC	26549025
XIST	miR-497	MACC1	Prognostic biomarker and therapeutic target	Promote	GC	27911852
MIR99AHG	miR577	FOXP1	Therapeutic target	Promote	GC	32874129
HIF1A-AS2	miR-429	PD-L1	Prognostic biomarker and therapeutic target	Promote	GC	33555514
LINC00184	miR-145	ANGPT2	Biomarker and therapeutic target	Promote	GC	33758610
DDX11-AS1	miR-326	IRS1	Prognostic biomarker and therapeutic target	Promote	GC	32271422
LOXL1-AS1	miR-708-5p	USF1	Prognostic biomarker	Promote	GC	31468594
GCMA	miR-124, miR-34a	Slug, Snail	Prognostic biomarker and therapeutic target	Promote	GC	32439864
PVT1	miR-30a	Snail	Therapeutic target	Promote	GC	32557622
HOTAIR	miR-1277-5p	COL5A1	Prognostic biomarker and therapeutic target	Promote	GC	32583079
SNHG6	miR-101-3p	ZEB1	Prognostic biomarker and therapeutic target	Promote	GC	28683446
LINC01133	miR-106a-3p	APC	Prognostic biomarker and therapeutic target	Suppress	GC	30134915
MT1JP	miR-92a-3p	FBXW7	Prognostic biomarker and therapeutic target	Suppress	GC	29720189
XIST	miR-185	TGF-β1	Prognostic biomarker	Promote	GC	29053187
UFC1	miR-498	Lin28b	Prognostic biomarker and therapeutic target	Promote	GC	29970131
SNHG11	miR-184	CDC25A	Prognostic biomarker	Promote	GC	33816469
LINC01503	miR-133a-5p	VIM	Prognostic biomarker	Promote	GCA	33200343
UICLM	miR-215	ZEB2	Prognostic biomarker and therapeutic target	Promote	CRC	29187907
LEF1-AS1	miR-489	DRAPH1	Prognostic biomarker and therapeutic target	Promote	CRC	32248974
MIR4435-2HG	miR-206	YAP1	Prognostic biomarker and therapeutic target	Promote	CRC	32154166
SLC30A10	miR-21c	APC	Prognostic biomarker and therapeutic target	Promote	CRC	32633358
MCF2L-AS1	miR-874-3p	CCNE1	Prognostic biomarker	Promote	CRC	33037865
HOTAIR	miR-211-5p	FLT-1	Prognostic biomarker	Promote	CRC	34470574
HOATIR	miR-214	ST6GAL1	Therapeutic target	Promote	CRC	31694696
LINC01296	miR-26a	GALNT3	Therapeutic target	Promote	CRC	30547804
NEAT1	miR-34a	SIRT1	Prognostic biomarker and therapeutic target	Promote	CRC	30312725
LUNAR1	miR-495-3p	MYCBP	Prognostic biomarker	Promote	CRC	33300052
H19	miR-194-5p	SIRT1	Biomarker of chemoresistance	Promote	CRC	30451820
SNHG6	miR-26a/b, miR-214	EZH2	Therapeutic target	Promote	CRC	30626446
CCMAlnc	miR-5001-5p	HES6	Prognostic biomarker and therapeutic target	Promote	CRC	33681178
SNHG6	miR-181a-5p	E2F5	Prognostic and therapeutic biomarker	Promote	CRC	30666158
NEAT1	miR-193a-3p	IL17RD	Potential marker	Promote	CRC	30407674
Lnc-HSD17B11-1:1	miR-338-3p	MACC1	Therapeutic target	Promote	CRC	32595704
RP11-51O6.1	miR-206	YAP1	Biomarker and therapeutic target	Promote	CRC	34038520
MALAT1	miR-106b-5p	SLAIN2	Prognostic biomarker	Promote	CRC	30797712
MEG3	miR-9	E-cadherin, FOXO1	Prognostic biomarker	Suppress	EC	28539329
EIF3J-AS1	miR-373-3p	AKT1	Prognostic biomarker and therapeutic target	Promote	EC	32811869
SNHG12	miR-195-5p	BCL9	Prognostic biomarker	Suppress	ESCC	32086782
ROR	miR-145	FSCN1	Prognostic biomarker	Promote	ESCC	29430188
**circRNA-mediated ceRNA crosstalk**						
circFGD4	miR-532-3p	APC	Prognostic biomarker and therapeutic target	Suppress	GC	32633323
circRHOBTB3	miR-654-3p	p21	Therapeutic target	Suppress	GC	31928527
circ-PRMT5	miR-145, miR-1304	MYC	Prognostic biomarker and therapeutic target	Promote	GC	31701767
circ-PTPDC1	miR-139-3p	ELK1	Prognostic biomarker	Promote	GC	34803498
circ_0110389	miR-127-5p, miR-136-5p	SORT1	Prognostic biomarker and therapeutic target	Promote	GC	34162830
circ-RanGAP1	miR-877-3p	VEGFA	Prognostic biomarker and therapeutic target	Promote	GC	31811909
circHECTD1	miR-137	PBX3	Prognostic biomarker	Promote	GC	34001137
circPDSS1	miR-186-5p	NEK2	Biomarker and therapeutic target	Promote	GC	30417526
ciRS-7	miR-7	NA	Prognostic biomarker and therapeutic target	Promote	GC	28608528
circTMEM87A	miR-142-5p	ULK1	Prognostic biomarker and therapeutic target	Promote	GC	33155080
circLMTK2	miR-150-5p	c-Myc	Prognostic predictor and therapeutic target	Promote	GC	31722712
circ-DCAF6	miR-1231, miR-1256	NA	Prognostic biomarker	Promote	GC	31226266
circTMC5	miR-361-3p	RABL6	Prognostic predictor and therapeutic target	Promote	GC	34296378
circ0005654	miR-363	sp1	Therapeutic target	Promote	GC	34499009
circUBE2Q2	miR-370-3p	STAT3	Prognostic biomarker	Promote	GC	34611143
circLARP4	miR-424-5p	LATS1	Prognostic biomarker	Suppress	GC	28893265
circ-ATAD1	miR-140-3p	YY1	Prognostic biomarker and therapeutic target	Promote	GC	32169278
circNHSL1	miR-1306-3p	SIX1	Prognostic biomarker and therapeutic target	Promote	GC	31438963
circEGFR	miR-106a-5p	DDX5	Therapeutic target	Promote	CRC	34320120
circ3823	miR-30c-5p	TCF7	Therapeutic target	Promote	CRC	34172072
circ_0026416	miR-346	NFIB	Therapeutic target	Promote	CRC	33061846
circ_0000372	miR-495	IL6	Prognostic biomarker and therapeutic target	Promote	CRC	33534412
circBANP	let-7d-5p	HMGA1	Biomarker and therapeutic target	Promote	CRC	33981828
circMBOAT2	miR-519d-3p	TROAP	Biomarker	Promote	CRC	32796815
ciRS-7	miR-7	EGFR, RAF1	Prognostic biomarker and therapeutic target	Promote	CRC	28174233
circHIPK3	miR-7	AK, IGF1R, EGFR, YY1	Prognostic biomarker and therapeutic target	Promote	CRC	29549306
circVAPA	miR-125a	CREB5	Therapeutic target	Promote	CRC	32256212
circHIPK3	miR-637	STAT3	Prognostic biomarker	Promote	CRC	31631038
circCAMSAP1	miR-328-5p	E2F1	Prognostic biomarker and therapeutic target	Promote	CRC	31951832
ciRS-7	miR-7	HOXB13	Prognostic marker and therapeutic target	Promote	ESCC	30082829

GC, Gastric cancer; GCA, Gastric cardia adenocarcinoma; CRC, colorectal cancer; EC, Esophageal cancer; ESCC, Esophageal squamous cell cancer.

Furthermore, increasing ceRNA players have emerged as potential therapeutic targets for GIC patients due to their critical roles in tumor progression (**Table 1**). For example, lncRNAs (e.g. HIF1A-AS2, GCMA and HOTAIR) and circRNAs (e.g. circ-RanGAP1, TMEM87A, circLMTK2 and circTMC5) implicated in GC metastasis by acting as ceRNAs, hold promise as potential therapeutic targets for GC patients (29–35). Besides, development of chemoresistance remains a primary obstacle for GIC treatment. It has been demonstrated that DDX11-AS1 can contribute to oxaliplatin resistance in GC by sponging miR-326, implying its therapeutic role (36). circHIPK3 and H19 have been reported to promote oxaliplatin and 5-FU resistance in CRC by mediating different ceRNA interactions, respectively (**Figure 1**) (37, 38). Those findings indicate that targeting circHIPK3 and H19 are also potential therapeutic strategies to inhibit chemoresistance in CRC.

Collectively, as pivotal factors mediating cancer pathogenesis, ceRNA players have emerged as promising prognostic biomarkers and attractive therapeutic targets in the clinical management of GICs.

## Prognostic Signatures Based on lncRNA/circRNA-Mediated ceRNA Network in GICs

As ceRNA networks connect the function of different RNA species, the characterization of cancer-specific ceRNA network may provide a valuable clue to systematically explore the potential role of RNA molecules in cancer pathogenesis. Therefore, a number of efforts have focused on construction of signatures based on lncRNA/circRNA-mediated ceRNA network in GICs (**Table 2**), illuminating new avenues to explore powerful prognostic biomarkers and therapeutic targets in the era of precision medicine.

**Table 2:** ceRNA network-based prognostic signatures in GICs.

Signature	Function	Included parameters	Performance		Cancer type	PMID
			Training dataset	Testing dataset		
**Signatures based on lncRNA-mediated ceRNA network**						
Gao et al.’s signature	Predicting OS	6 lncRNA	NA	NA	CC	33836755
Guo et al.’s signature	Predicting OS	2 lncRNAs, 1 miRNA, and 3 genes	AUC of 0.634 at 1 year, 0.68 at 3 years, and 0.66 at 5 years	AUC of 0.775 at 1 year, 0.836 at 3 years, and 0.804 at 5 years in validation 1 dataset; AUC of 0.586 at 1 year, 0.62 at 3 years, and 0.632 at 5 years in validation 2 dataset	CRC	34276767
Huang et al.’s signature	Predicting OS	5 lncRNAs	AUC of 0.850	NA	CC	31448228
Li et al.’s signature	Predicting OS	3 lncRNAs	NA	NA	CC	33858429
Li et al.’s signature	Predicting OS	7 genes	AUC of 0.720 at 1 year, 0.741 at 3 years, and 0.714 at 5 years	NA	CRAC	34692502
Liu et al.’s signature	Predicting OS	3 lncRNAs	AUC of 0.716 at 5 years	AUC of 0.649 at 5 years	CRC	33302562
Peng et al.’s signature	Predicting OS	8 lncRNAs	AUC of 0.738 at 1 year, 0.746 at 3 years and 0.784 at 5 years	NA	CRC	34458145
Qian et al.’s signature	Predicting OS	3 genes	NA	NA	CRC	29916526
Xu et al.’s signature	Predicting OS	1 lncRNA, 2 miRNAs, and 4 genes	AUC of 0.698 at 1 year, 0.739 at 3 years and 0.781 at 5 years	NA	CC	34692670
Yang et al.’s signature	Predicting OS	7 genes	AUC of 0.746 at 1 year, 0.744 at 3 years and 0.772 at 5 years	NA	CC	31612869
Yang et al.’s signature	Predicting OS	4 lncRNAs	AUC of 0.628	AUC of 0.649	CRC	32256018
Zhang et al.’s signature	Predicting OS and DFS	5 lncRNAs	AUC of 0.675 for OS and 0.690 for DFS at 5 years	AUC of 0.695	CRC	30714675
Zhang et al.’s signature	Predicting chemotherapy resistance and survival	8 lncRNAs	AUC of 0.87 in predicting the FOLFOX chemotherapy response in metastatic CRC patients	NA	CRC	33585448
Zhang et al.’s signature	Predicting OS	15 genes	C-index of 0.817 at 1 year, 0.838 at 3 years and 0.825 at 5 years	C-index of 0.773 at 1 year, 0.824 at 3 years and 0.801 at 5 years	CRC	31796117
Wei et al.’s signature	Predicting OS	1 lncRNA and 1 miRNA	AUC of 0.71 at 1 year, 0.79 at 3 years and 0.97 at 5 years	NA	RC	34350117
Li et al’s signature	Predicting OS	3 lncRNAs	AUC of 0.639 at 3 years, AUC of 0.685 at 5 years	NA	EC	33381546
Zhang et al’s signature	Predicting OS	6 lncRNA	AUC of 0.686	NA	ESCC	34603485
Chen et al’s signature	Predicting recurrence	4 lncRNAs	AUC of 0.936	AUC of 0.827 in validation 1 dataset; AUC of 0.882 in validation 2 dataset	GC	33869776
Mao et al.’s signature	Predicting OS	3 lncRNAs and 3 mRNAs	AUC of 0.699 at 3 years, 0.739 at 4 years, 0.801 at 5 years, 0.766 at 6 years and 0.853 at 7 years	AUC of 0.809 at 3 years, AUC of 0.820 at 4 years	GA	33188157
Qi et al.’s signature	Predicting OS	2 lncRNAs	AUC of 0.614	NA	GC	31923354
Zhang et al.’s signature	Predicting OS	2 lncRNAs	AUC of 0.651 at 3 years	AUC of 0.615 at 3 years	GC	34603561
**Signatures based on circRNA-mediated ceRNA network**						
Song et al.’s signature	Predicting OS	7 genes	AUC of 0.701 at 3 years and 0.728 at 5 years	NA	CRC	32582276
Wang et al.’s signature	Predicting OS	8 genes	AUC of 0.77 at 1 year, 0.92 at 3 years and 0.78 at 5 years	NA	EAC	33376353
Han et al.’s signature	Predicting OS	11 genes	AUC of 0.741	NA	GC	33514881
Li et al.’s signature	Predicting OS	3 genes	NA	NA	GC	33969120

CRC, colorectal cancer; CC, colon cancer; CRAC, colorectal adenocarcinoma; RC, rectal cancer; EC, esophageal cancer; ESCC, esophageal squamous cell carcinoma; GC, gastric cancer; GA, gastric adenocarcinoma; EAC, esophageal adenocarcinoma; OS, overall survival; DFS, disease-free survival. NA, Not available.

### ceRNA Network-Based Prognostic Signatures in GC

GC is a serious health problem throughout the world with high morbidity and mortality. Due to the lack of early disease-specific symptoms, most GC patients are diagnosed at advanced stages with unsatisfactory prognosis. Since survival probability is a major concern for cancer patients, signatures developed based on lncRNA-mediated ceRNA network are usually used to predict overall survival (OS) of GC patients (**Table 2**). For example, based on integrative analysis of the GC-specific ceRNA network, Zhang et al. (39) established a two-lncRNA signature consisting of LINC01644 and LINC01697 as a prognostic biomarker for survival prediction of GC patients. Functionally, knockdown of LINC01644 or LINC01697 could inhibit GC cell proliferation. Similarly, Li *et al.* (40) investigated the clinical significance of genes within the circRNA-mediated ceRNA network and further build a three-gene risk model for predicting OS in GC patients. The findings not only unravel the regulatory mechanisms of circRNAs, but also guide individualized management.

Furthermore, as principal causes of cancer-related death, metastasis and recurrence have long been considered as critical events influencing prognosis and treatment effect of cancer patients. Understanding the risk of metastasis and recurrence is critical for the success of personalized cancer therapy. Therefore, prognostic signatures based on lncRNA/circRNA-mediated ceRNA network are increasingly developed to predict metastasis or recurrence of GC patients, thus helping to optimize clinical treatment and management. For example, Chen *et al.* (41) successfully established a four-lncRNA signature to predict prognosis and distinguish recurrence risk of GC patients with robust performance.

### ceRNA Network-Based Prognostic Signatures in CRC

CRC remains the most common gastrointestinal tract malignancy, ranking second for cancer-related mortality globally. Emerging evidence reveals that dysregulation of ceRNA crosstalks is closely involved in the pathological biology of CRC, making ceRNA network-based prognostic signature a promising tool for guiding personalized therapy (**Table 2**). For example, based on metastasis-associated ceRNA network, Liu et al. (42) developed a three-lncRNA signature including LINC00114, LINC00261, and HOTAIR, and proved its powerful prognostic value for CRC patients. Functionally, LINC00114 can suppress CRC cell proliferation and migration by sponging miR-135a.

Notably, biological process or pathway (e.g. immune, autophagy and fatty acid metabolism)-specific ceRNA networks are widely used to establish prognostic signatures in CRC. First, given the close association between immune infiltration level and clinical outcome in cancers, unraveling cancer-specific ceRNA network tightly associated with immune regulation can facilitate the development of prognostic signatures. For example, Song et al. (43) developed a novel signature consisting of seven immune-related genes based on circRNA-mediated ceRNA network, and proved that the immune-related signature can predict OS of CRC patients with high accuracy. Second, autophagy is a conserved intracellular degradative process, which plays critical roles in maintaining cellular metabolism, homeostasis and survival. Dysregulation of the autophagy process has been shown to be closely related to the pathogenesis of various cancers. By integrating the reported autophagy-related genes and the experimentally verified miRNA-mRNA and miRNA-lncRNA interactions, Qian et al. (44) established an autophagy-related ceRNA network and further constructed multi-gene models for OS prediction in colon cancer and rectal cancer, respectively. Besides, perturbation of fatty acid metabolism has recently been recognized as a hallmark of cancer. Peng et al. (45) successfully built a prognostic signature containing eight fatty acid metabolism-related lncRNAs identified from the ceRNA network, and found that the fatty acid metabolism-related lncRNA signature can predict OS in CRC patients with high accuracy (AUC>0.7), which is superior to traditional clinical factors, such as age and stage. Therefore, process or pathway-related ceRNA network has provided a useful tool for constructing prognostic signatures in CRC.

### ceRNA Network-Based Prognostic Signatures in EC

EC is also known as one of the most commonly diagnosed gastrointestinal tumors with approximately 604,100 new cases annually (1). Despite technological improvement achieved in diagnosis and treatment, the 5-year survival rate of EC patients is below 20% (46), indicating poor prognosis. Recently, increasing studies have shown that lncRNAs participate in the post-transcriptional regulation of EC carcinogenesis through the ceRNA mechanism, exhibiting prognostic potential (**Table 2**). For example, based on integrated analysis of lncRNA-mediated ceRNA network, Li et al. (47) and Zhang et al. (48) successfully developed a novel three-lncRNA and six-lncRNA panel with prognostic value for EC patients by employing multiple Cox regression analysis, respectively. Similarly, Wang et al. (49) established a novel eight-gene signature as an independent prognostic factor for predicting the OS of patients with esophageal adenocarcinoma (EAC).

## Computational Establishment of ceRNA Network-Based Prognostic Signature

**Figure 2:**
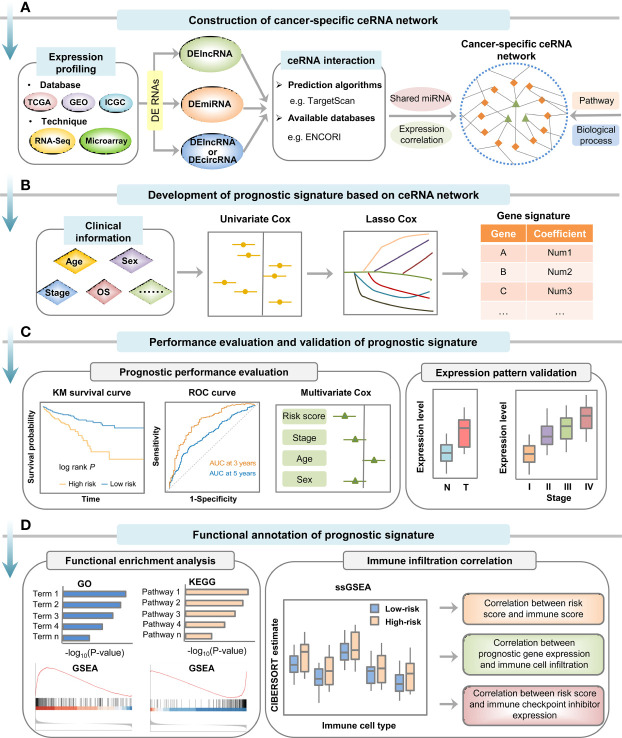
Computational strategy for construction, validation and functional annotation of ceRNA network-based prognostic signature in cancer. **(A)** Cancer-specific ceRNA network was constructed based on expression and interaction information. **(B)** Prognostic signature was developed by employing univariate Cox regression analysis and LASSO Cox regression analysis. **(C)** Prognostic performance of the signature should be evaluated and validated by Kaplan-Meier survival curve analysis, time-dependent ROC curve analysis and multivariate Cox regression analysis, and the expression pattern of genes that make up the model can be verified in other independent datasets. **(D)** Biological role of the prognostic signature could be investigated by functional enrichment analysis and immune infiltration analysis.

Compelling functional studies have demonstrated that dysregulation of ceRNA crosstalk can contribute to tumor progression by affecting a variety of signaling pathways involved in cancer hallmarks, paving the way for the establishment of novel prognostic signatures in various cancer types. Collectively, the computational strategy for developing ceRNA network-driven signature primarily consists of a series of steps, including cancer-specific ceRNA network construction, risk model construction and validation, and functional annotation (**Figure 2**).

### Cancer-Specific ceRNA Network Construction

According to the ceRNA theory, endogenous RNAs competitively bind to shared miRNAs, thereby regulating mutual expression. Therefore, the computational methods used to identify ceRNA interactions mainly rely on complementary base pairing and expression correlation between miRNA and its targets (50).

With increasing amounts of cancer data becoming available at public databases (e.g. TCGA and GEO), construction of ceRNA networks based on transcriptome analysis has been extensively employed to investigate key ceRNA crosstalks in multiple cancer types (50, 51). In this method, differential expression analysis was commonly performed to identify RNA molecules implicated in cancer-related processes, such as cancer initiation, progression or metastasis. Meanwhile, miRNA-target pairs were usually recognized by prediction algorithms (e.g. miRanda (52), TargetScan (53), RNAhybrid (54), RNA22 (55), etc.) and available databases collecting predictive or experimental information (e.g. miRCode (56), starBase v2.0 (57), miRTarBase (58), DIANA-LncBase v3 (59), CircInteractome (60), etc.). Following evaluation of miRNA regulatory similarity, expression correlation between each putative ceRNA pair was frequently evaluated by Pearson correlation coefficients. Besides, several R/Bioconductor packages, such as Sparse Partial correlation ON Gene Expression (SPONGE) and miRspongeR, are available for fast identification of ceRNA crosstalks and construction of ceRNA networks (61, 62). Then, based on the ceRNA triplets composed by differentially expressed RNAs as well as biological process or pathway information, cancer-specific ceRNA network can be constructed and visualized *via* Cytoscape software or R packages (**Figure 2A**) (63).

In addition to the above strategy, extensive efforts have been made to develop novel approaches for prediction of miRNA-mediated ceRNA crosstalks and construction of ceRNA networks. For example, Chiu et al. (64) designed an integrative framework named Cupid for context-specific prediction of both miRNA-target and ceRNA interactions simultaneously based on sequence and expression information. Helwak et al. (65) developed a crosslinking, ligation, and sequencing of hybrids (CLASH)-based method for high-throughput identification of miRNA-target interaction directly. Furthermore, considering the influence of kinetic parameters on miRNA-mediated interaction between ceRNAs, multiple computational/mathematical models have been developed to study dynamics of the ceRNA crosstalk in diverse biological settings (66). For example, Bosia et al. (67) proposed a stochastic model to explore the equilibrium and non-equilibrium characteristics of ceRNA networks based on the miRNA-target titration mechanism. Chiu et al. (68) proposed a kinetic model for ceRNA regulation that accounts for the influence of co-regulation by miRNAs with multiple targets and found that ceRNA interaction is strongly affected by the abundance of miRNA mediators and the number of miRNA targets. Therefore, increasing breakthroughs have been achieved in the development of computational approaches for ceRNA network construction.

### Construction and Validation of Prognostic Signatures

Based on cancer-specific ceRNA networks, signatures can incorporate multiple types or a single type of RNAs. Among them, lncRNA was the most reported type, so the present study takes it as an example to introduce signature construction and verification methods. First, the prognostic value of lncRNAs involved in the cancer-specific ceRNA network can be evaluated by univariate Cox regression analysis of the association between lncRNA expression level and patient survival time (69). Then, lncRNA-related prognostic signature was commonly established by performing LASSO Cox regression analysis or multivariate Cox regression analysis (70). The risk score for each patient was calculated based on the coefficient and normalized expression value of each lncRNA included in the signature (**Figure 2B**). Furthermore, multivariate Cox regression analysis could also be employed to test whether the lncRNA-related signature was an independent predictor for patient survival (**Figure 2C**) (71). For example, based on comprehensive analysis of ceRNA network, Mao et al. (70) established a six-lncRNA signature for recurrent prognosis prediction of patients with colon adenocarcinoma by using LASSO Cox regression model. Similarly, Tao et al. (72) developed a vascular invasion-related lncRNA signature to predict the OS of hepatocellular carcinoma patients by utilizing univariate, LASSO and multivariate Cox regression analyses.

To evaluate the robustness of the constructed signature for prognosis prediction, the patients in both training and testing datasets were usually divided into high- and low-risk subgroups, followed by Kaplan-Meier survival curve analysis and time-dependent ROC curve analysis (**Figure 2C**). Indeed, the prognostic performance of most ceRNA network-based signatures has been evaluated and/or validated through Kaplan-Meier survival curve and ROC curve analyses (42, 73, 74).

### Functional Annotation of Prognostic Signatures

The common functional enrichment analyses, such as Gene Ontology (GO), Kyoto Encyclopedia of Genes and Genomes (KEGG) and Gene Set Enrichment Analysis (GSEA), could be used to explore the potential functions of the established lncRNA/circRNA-related signature. Generally, given the principle that co-expressed ncRNAs and mRNAs might share biological roles, GO and KEGG enrichment analyses were frequently performed on the genes co-expressed with model ncRNAs identified by computational methods. Besides, based on the Molecular Signatures Database (MSigDB), GSEA can also be utilized to explore the biological function of prognostic signatures (**Figure 2D**). For example, Liu et al. (42) found that the key lncRNAs that constitute the prognostic model were implicated in CRC tumorigenesis through GO and KEGG enrichment analyses on the co-expressed genes. Based on GSEA results, Chen et al. (69) found that the constructed eleven-lncRNA prognostic signature was involved in immune-related processes of hepatocellular carcinoma.

Furthermore, given the close link between immune and cancer pathogenesis, single-sample Gene Set Enrichment Analysis (ssGSEA) could be conducted to investigate the relationship between prognostic signature and immune status by calculating infiltration scores of distinct immune cell types based on the abundance of immune-related marker genes (**Figure 2D**) (75). Besides, immune infiltration correlation analyses, such as correlation between signature-based risk score and immune score, correlation between prognostic gene expression level and immune cell infiltration, and correlation between signature-based risk score and immune checkpoint inhibitor expression level, can be used to investigate the biological role of the established signature (**Figure 2D**) (43, 69, 76).

## Discussion and Perspective

In view of the complex and heterogeneous characteristic of GICs, satisfactory prognostic evaluation of patients is difficult to accomplish. With the constant effort and advances in gene expression regulation, accumulating evidence has proved that both coding and non-coding RNAs (e.g. mRNA, lncRNA, and circRNA) hold the power to communicate with each other through a miRNA-mediated ceRNA mechanism (9). Given the potential roles in cancer pathogenesis and progression, the translational significance of ceRNA molecules has recently attracted increasing attention in GICs. It should be noted that a single miRNA can bind to multiple different targets according to the mechanism of action of miRNA. The diversity of miRNA target genes determines that the ceRNA crosstalk does not work alone, but through forming a coordinated large interaction networks where significative crosstalk could take place between distant RNAs under physiological and pathological conditions. For example, Rzepiela et al. (77) discovered the hierarchical response dynamics of distinct miRNA targets to miRNA induction by combining mathematical modeling with single-cell mRNA profiling, promoting our understanding of the complexity of ceRNA networks. Miotto et al. (78) found that despite the weakness of individual ceRNA crosstalk, extended miRNA-RNA networks could facilitate the integration of a huge number of interactions, leading to significant system-level effect. Besides, Chiu et al. (68) also highlighted the impact of the number and abundance of titrated microRNA species on ceRNA regulation. Therefore, the paradigm of ceRNA biomarker discovery is gradually shifting from individual ceRNA identification and validation toward the exploration of interaction relationship in ceRNA networks under a systematic framework of gene regulation.

Currently, increasing researches towards ceRNA networks in GICs has not only enhanced our understanding of ceRNA-mediated GICs pathogenesis, but also paved the way for developing novel prognostic biomarkers and therapeutic targets for GICs patients (79). Indeed, a large number of studies have identified prognostic signatures that predict the OS, metastasis or recurrence of patients with GC, CRC or EC through an integrated analysis of cancer-related ceRNA network (41, 80, 81). However, most of those signatures have not reached the criteria of well-validated effective prognostic models that could improve risk stratification and therapeutic decision making in pre-clinical and clinical practice. On one hand, ceRNA network-based prognostic signatures were commonly established by employing expression profiling datasets collected in public databases, such as TCGA or GEO. Their prognostic value needs to be confirmed in independent large and diverse population cohorts with GIC. On the other hand, the major obstacle for clinical application of ceRNA network-based prognostic signatures are largely due to the lack of a clear understanding of their functional roles in tumorigenesis, and the specific downstream signaling pathways and targets that they regulate. Therefore, although our understanding for the functions of ceRNA crosstalks in GICs continues to deepen, there is still much to explore to bridge the gap between theoretical research and clinical translation.

As different types of GICs, such as GC, CRC and EC, possess varying clinical manifestations, course and outcomes, the reported prognostic signatures are commonly constructed based on cancer-specific ceRNA networks. Accordingly, based on the published literatures, we found no evidence that any of the reported ceRNA network-based prognostic signatures are applicable to multiple cancer types. In fact, it is challenging to create a general ceRNA signature in multiple cancer types, as ceRNA interactions mainly depend on the abundance of free RNAs, and the expression of genes required for specific functions varies widely in distinct tissues (82). However, ceRNA interactions explain that even a slight amount change in a certain transcript can affect the abundance of other transcripts in indirect ceRNA:miRNA:ceRNA interactions. Therefore, large-scale analysis is needed to explore ceRNA functions. In addition, due to the pivotal role of certain process or pathway involved in carcinogenesis, process or pathway-specific ceRNA network provides novel strategies for powerful prognostic signature building.

Single-cell RNA sequencing technologies have revolutionized the field of cancer biology as they provide unprecedented opportunities to reveal the properties of distinct cell populations at single-cell resolution (83). Considering the impact of intratumoral heterogeneity on clinical practice of GICs, construction of cellular-specific ceRNA networks will deepen the quantitative understanding of cancer pathogenesis and further promote the development of precision medicine (84). Recently, the database of cellular-specific lncRNA-mediated ceRNA networks, LnCeCell, has been constructed based on single-cell RNA sequencing datasets and published literature. It collected ceRNA interactions from a large number of cells across 25 cancer types, facilitating the decoding of ceRNA regulations at single-cell level (85). Therefore, with the advance of single cell expression profiling approaches, cellular-specific ceRNA networks provide a new route to establish prognostic signatures in the future.

In summary, although the field of ceRNA network-based prognostic signatures is still in its infancy, we are currently witnessing their translational and clinical significance in multiple GICs and other diseases. With further convincing validations and functional explorations, those signatures will be helpful to optimize individualized management and treatment as well as to improve clinical outcomes of patients with GIC in the era of personalized medicine.

## Author Contributions

BS and XQ designed the review. XQ collected the related data and drafted the manuscript. XC and YZ revised the tables and figures. BS, XQ, JC, and BN revised the manuscript. All authors read and approved the final manuscript.

## Funding

This work was supported by National Natural Science Foundation of China (Grant No. 31900490).

## Conflict of Interest

The authors declare that the research was conducted in the absence of any commercial or financial relationships that could be construed as a potential conflict of interest.

## Publisher’s Note

All claims expressed in this article are solely those of the authors and do not necessarily represent those of their affiliated organizations, or those of the publisher, the editors and the reviewers. Any product that may be evaluated in this article, or claim that may be made by its manufacturer, is not guaranteed or endorsed by the publisher.

## Abbreviations

GIC, Gastrointestinal cancers; ncRNA, non-coding RNA; ceRNA, competing endogenous RNA; lncRNA, long non-coding RNA; circRNA, circular RNA; GC, gastric cancer; CRC, colorectal cancer; EC, esophagus cancer; TNM, tumor-node-metastasis; miRNA, microRNA; MREs, miRNA-response elements; SPONGE, Sparse Partial correlation ON Gene Expression; CLASH, crosslinking, ligation, and sequencing of hybrids; SNHG, small nucleolar RNA host gene; OS, overall survival; EMT, epithelial-mesenchymal transition; EAC, esophageal adenocarcinoma; GO, Gene Ontology; KEGG, Kyoto Encyclopedia of Genes and Genomes; GSEA, Gene Set Enrichment Analysis; MSigDB, Molecular Signatures Database; ssGSEA, single-sample Gene Set Enrichment Analysis.
